# Comparative Analysis of Primary and Monovalent Booster SARS-CoV-2 Vaccination Coverage in Adults with and without HIV in Catalonia, Spain

**DOI:** 10.3390/vaccines12010044

**Published:** 2023-12-30

**Authors:** Daniel Kwakye Nomah, Juliana Reyes-Urueña, Lucía Alonso, Yesika Díaz, Sergio Moreno-Fornés, Jordi Aceiton, Andreu Bruguera, Raquel Martín-Iguacel, Arkaitz Imaz, Maria del Mar Gutierrez, Ramón W. Román, Paula Suanzes, Juan Ambrosioni, Jordi Casabona, Jose M. Miro, Josep M. Llibre, the PISCIS Study Group

**Affiliations:** 1 Center for Epidemiological Studies of Sexually Transmitted Diseases and HIV/AIDS in Catalonia (CEEISCAT), Department of Health, Government of Catalunya, 08916 Badalona, Spain; juliana.reyes81@gmail.com (J.R.-U.); lalonso@lluita.org (L.A.); ydiazr@iconcologia.net (Y.D.); smorenof@iconcologia.net (S.M.-F.); jaceiton@iconcologia.net (J.A.); abruguerar@iconcologia.net (A.B.); raquel@bisaurin.org (R.M.-I.); jcasabona@iconcologia.net (J.C.); 2 Germans Trias i Pujol Research Institute (IGTP), 08916 Badalona, Spain; 3 CIBER Epidemiologia y Salud Pública (CIBERESP), 08003 Barcelona, Spain; 4 Departament de Pediatria, d’Obstetrícia i Ginecologia i de Medicina Preventiva i de Salut Publica, Universitat Autònoma de Barcelona, 08193 Bellaterra, Spain; 5 Department of Infectious Diseases, Odense University Hospital, 5000 Odense, Denmark; 6 Department of Infectious Diseases, Hospital Universitari de Bellvitge-(IDIBELL), 08907 L’Hospitalet de Llobregat, Spain; aimaz@bellvitgehospital.cat; 7 Hospital de la Santa Creu i Sant Pau, 08041 Barcelona, Spain; mgutierrezma@santpau.cat; 8 Agència de Qualitat i Avaluació Sanitàries de Catalunya, 08005 Barcelona, Spain; ramon.roman@gencat.cat; 9 Hospital Universitari Vall d’Hebron, Vall d’Hebron Research Institute (VHIR), 08035 Barcelona, Spain; paula.suanzes@vhir.org; 10 Hospital Clínic-Institut d’Investigacions Biomèdiques August Pi i Sunyer (IDIBAPS), University of Barcelona, 08036 Barcelona, Spain; ambrosioni@clinic.cat (J.A.); jmmiro@ub.edu (J.M.M.); 11 CIBERINFEC, Instituto de Salud Carlos III, 28029 Madrid, Spain; 12 Hospital Universitari Germans Trias i Pujol, 08916 Badalona, Spain; jmllibre@lluita.org

**Keywords:** HIV, SARS-CoV-2, COVID-19, vaccination, booster doses

## Abstract

People with HIV (PWH) may be more susceptible to SARS-CoV-2 infection and worse clinical outcomes. We investigated the disparity in SARS-CoV-2 vaccination coverage between PWH and those without HIV (PWoH) in Catalonia, Spain, assessing primary and monovalent booster vaccination coverage from December 2021 to July 2022. The vaccines administered were BNT162, ChAdOx1-S, mRNA-127, and Ad26.COV2.S. Using a 1:10 ratio of PWH to PWoH based on sex, age, and socioeconomic deprivation, the analysis included 201,630 individuals (183,300 PWoH and 18,330 PWH). Despite a higher prevalence of comorbidities, PWH exhibited lower rates of complete primary vaccination (78.2% vs. 81.8%, *p* < 0.001) but surpassed PWoH in booster coverage (68.5% vs. 63.1%, *p* < 0.001). Notably, complete vaccination rates were lower among PWH with CD4 <200 cells/μL, detectable HIV viremia, and migrants compared to PWoH (*p* < 0.001, all). However, PWH with CD4 < 200 cells/μL received more boosters (*p* < 0.001). In multivariable logistic regression analysis of the overall population, a prior SARS-CoV-2 diagnosis, HIV status, migrants, and mild-to-severe socioeconomic deprivation were associated with lower primary vaccination coverage, reflecting barriers to healthcare and vaccine access. However, booster vaccination was higher among PWH. Targeted interventions are needed to improve vaccine coverage and address hesitancy in vulnerable populations.

## 1. Introduction

Remarkable scientific and governmental investments have been made to develop multiple vaccine candidates against severe acute respiratory syndrome coronavirus 2 (SARS-CoV-2) in an unprecedented time [1]. These vaccines have proven to be an effective and viable approach to combat the ongoing pandemic and mitigate its socioeconomic and health impacts. The European Medicines Agency (EMA) has authorized eight vaccines for use in the European Union [2]. As of 22 November 2023, over 13.5 billion SARS-CoV-2 vaccine doses had been administered across 184 countries, making it the largest vaccination campaign in human history [3]. 

People with HIV (PWH) may be more susceptible to SARS-CoV-2 infection and face worse outcomes. According to a report from the World Health Organization (WHO), PWH have a 30% higher risk of mortality from COVID-19 after hospital admission compared to people without HIV (PWoH) [4]. Additionally, PWH may face poorer COVID-19 outcomes due to other social determinants of health, chronic comorbid conditions, and poor HIV control [5,6]. As a result, many countries prioritized PWH for vaccine eligibility. 

Existing evidence demonstrates that SARS-CoV-2 vaccines offer protection against COVID-19 by effectively reducing symptomatic infections and severe outcomes [7]. However, vaccine hesitancy among certain sub-populations [8,9] and the decline in IgG antibody levels after SARS-CoV-2 infection or vaccination [10] have hindered the full potential of vaccine protection. The emergence of SARS-CoV-2 variants with increased transmissibility and the ability to evade vaccine-induced immunity [11] is also a cause for concern. The uncertainties regarding the duration of vaccine protection and the impact of new variants underscored the importance of booster vaccinations [12]. Studies have shown that booster doses significantly decrease the risk of severe COVID-19 [13,14]. There are recommendations to administer booster doses to PWH with advanced immunosuppression or untreated HIV infection due to their increased risk of severe COVID-19 illness and potentially weaker immune response to SARS-CoV-2 vaccination [15]. In addition to their increased vulnerability, PWH may encounter barriers that limit their access to the crucial SARS-CoV-2 vaccinations [16]. 

Research on vaccination coverage among PWH is limited and lacks comparison with a matched sample from the general population [17,18]. Since vaccination strategies in many countries prioritize the public based on factors such as the nature of their jobs, age, presence of comorbidities, and other risk factors for adverse COVID-19 outcomes, matched studies are essential to assess the equity and effectiveness of current vaccination strategies, identify under-vaccinated groups, and provide valuable insights for future pandemics. The objective of this report is to compare primary and booster monovalent vaccination coverage among PWH with a well-matched representative sample of PWoH in Catalonia, Spain, and to identify subpopulations with low vaccination uptake to inform public health policies on ongoing vaccination strategies and future vaccination campaigns. 

## 2. Materials and Methods

### 2.1. Study Design and Population

We conducted a retrospective cohort study using data from the prospective PISCIS cohort linked with integrated healthcare, clinical, and surveillance registries through the Data Analysis Program for Research and Innovation in Health (PADRIS) [19] to obtain information on vaccination. PISCIS is an ongoing, population-based, longitudinal, systematic, prospective, and multicentre HIV cohort study of individuals receiving care in Catalonia and the Balearic Islands, Spain. Details of the cohort have been described elsewhere [20]. For the purposes of this study, we used participants receiving care in the 16 PISCIS hospitals in Catalonia, representing approximately 84% of all PLWH in the region.

People with HIV were matched 1:10 to HIV-negative individuals from the general population in Catalonia for sex at birth, 5-year age group, and socioeconomic deprivation using exact matching. The socioeconomic index is generated by the Catalan government based on the basic health area of residence (ABS, abbreviation in Catalan) to determine the socioeconomic levels of Catalonia residents [21] and takes into account the following indicators: the proportion of manual workers, the proportion of residents with low education levels, the proportion with low incomes, the rate of premature mortality, and the rate of avoidable hospitalization [21]. 

We excluded PWH who were not alive as of 27 December 2020, the day the vaccination campaign began in Spain, as well as those not in active clinical follow-up (those who have not used healthcare services for at least 12 months) to ensure the accurate estimation of vaccine coverage. HIV-negative individuals were classified as such if there was no record of HIV infection based on the absence of HIV International Classification of Diseases (ICD) codes. The study period was from 27 December 2020 to 19 July 2022.

### 2.2. Outcomes

We defined complete primary vaccination according to the criteria set by the WHO: (a) two doses of the BNT162 (Pfizer), mRNA-1273 (Moderna), or ChAdOx1-S (Oxford/AstraZeneca) vaccines; or (b) a single dose of Janssen Ad26.COV2.S [22]. Incomplete vaccination was defined as receiving only a single dose of the BNT162 (Pfizer), mRNA-1273 (Moderna), or ChAdOx1-S (Oxford/AstraZeneca) vaccines. Booster vaccinations were defined as any additional doses administered after completing the primary vaccination series [22]. 

### 2.3. Covariates

The sociodemographic covariates included age as of 1 January 2021, sex assigned at birth, country of origin classified as Spanish or non-Spanish, socioeconomic deprivation grouped into least deprivation, mild deprivation, or moderate-to-severe deprivation. The COVID-19-associated variables included are: history of SARS-CoV-2 diagnosis defined as a positive nucleic acid amplification test (NAAT) and/or antigen detection from respiratory samples [23]. Comorbidity covariates included the most prevalent conditions in the PWH population cohort: chronic respiratory disease, cardiovascular disease, chronic kidney or liver disease, neuropsychiatric conditions, diabetes, cancer, hypertension, obesity, and autoimmune disease. Comorbidity data were extracted using the ICD-10 codes (Appendix A). Among PWH, additional data were collected on years since HIV diagnosis, HIV transmission risk group (people who inject drugs [PWID], men who have sex with men [MSM], male heterosexual, female sexual transmission, and others), antiretroviral therapy (ART) reception, most recent CD4 cell count (categorized as <200 cells/μL, 200–499 cells/μL, and ≥500 cells/μL), and HIV plasma RNA viral load categorized as detectable and undetectable (<50 copies/mL).

### 2.4. Statistical Analysis 

We described the distribution of sociodemographic and clinical variables between PWH and PWoH to determine differences between the two populations. We used multivariable logistic regression models to assess the factors associated with complete vaccine reception and booster vaccinations, providing adjusted odds ratios (aOR) with 95% confidence intervals (95% CIs). The models were adjusted for age, sex, country of origin, socioeconomic deprivation, prior SARS-CoV-2 diagnosis, number of comorbidities, and HIV status. We calculated the cumulative incidence of complete vaccine reception and booster doses using Kaplan–Meier techniques from January 2021 to April 2022. We stratified the vaccine coverage analysis by HIV status, and among PWH, by country of origin (Spanish and non-Spanish), CD4 cell count categories, and HIV plasma RNA viral load. Log-rank tests were calculated to estimate the differences in cumulative vaccination coverage. We conducted subgroup analysis to investigate the factors associated with complete vaccine reception and booster vaccinations in both groups (Supplementary Material). We performed all analyses with R version 4.1.3 (R Project for Statistical Computing). A 2-sided *p*-value of <0.05 was considered statistically significant. 

### 2.5. Ethics

The Institutional Review Board of Germans Trias i Pujol Hospital in Badalona, Spain approved the PISCIS cohort study. Patient-level information obtained from PADRIS was anonymized and deidentified before analysis. This study followed the Strengthening the Reporting of Observational studies in Epidemiology (STROBE) guidelines.

## 3. Results

### 3.1. Baseline Characteristics of Study Population

A total of 18,330 PWH were matched to 183,300 PWoH in a ratio of 1:10. There were no differences in sex, age, and socioeconomic deprivation between the two groups. However, significant differences were observed regarding country of origin (*p* < 0.001), number of comorbidities (*p* < 0.001), and previous SARS-CoV-2 diagnosis (*p* < 0.001) between the two groups (Table 1).

Among the PWH, 15,062 individuals (82.2%) were male, and the majority (81.1%) fell within the age range of 31–60 years. The most common HIV acquisition risk group was MSM, accounting for 53.3%. The median (interquartile range [IQR]) CD4 cell count was 680 (486–908) cells/μL, with 627 PWH (3.4%) having a CD4 cell count below 200 cells/μL. The median (IQR) CD4/CD8 ratio was 0.85 (0.57–1.2), and 14,404 PWH (78.6%) had undetectable HIV RNA viremia (Table 1).

### 3.2. Vaccination Coverage 

Among the 201,630 individuals included in the study, 81.4% had received complete primary vaccination, while 63.5% had received booster doses. People with HIV had lower rates of complete vaccination compared to those without HIV (78.2% vs. 81.8%, *p* < 0.001). However, PWH had higher coverage of booster doses compared to the non-HIV group (68.5% vs. 63.1%, *p* < 0.001). The median duration in months between the primary vaccination series and the reception of a booster was similar in both groups at 6.4 months (IQR 6.0–7.1). Regarding the types of vaccines administered, the majority of study participants received the BNT162 BioNTech/Pfizer vaccine for the primary vaccination series (61.5%). However, for booster doses, the mRNA-1273 Moderna vaccine was more commonly administered (86.5%). The general HIV-negative population was more frequently vaccinated with BNT162 BioNTech/Pfizer; however, PWH vaccinated at hospitals received the mRNA-1273 Moderna as their primary dose (Table 2).

### 3.3. Factors Associated with Vaccine Coverage

In the overall population, a multivariable logistic regression analysis, adjusted for all potential confounders, revealed that PWH were less likely to receive the complete primary vaccine compared to PWoH (aOR 0.86; 95% CI 0.82–0.89). Other factors associated with lower odds of receiving the complete primary vaccine included non-Spanish origin (aOR 0.39; 95% CI 0.38–0.40), mild socioeconomic deprivation (aOR 0.87; 95% CI 0.84–0.90), moderate-to-severe socioeconomic deprivation (aOR 0.87; 95% CI 0.85–0.90), and a previous SARS-CoV-2 diagnosis (aOR 0.20; 95% CI 0.19–0.20) (Table 3).

Regarding booster vaccination, similar associations were observed. Non-Spanish origin (aOR 0.75; 95% CI 0.73–0.77), mild socioeconomic deprivation (aOR 0.80; 95% CI 0.78–0.83), moderate-to-severe socioeconomic deprivation (aOR 0.77; 95% CI 0.75–0.79), and a previous SARS-CoV-2 diagnosis (aOR 0.24; 95% CI 0.23–0.25) were all associated with lower odds of receiving booster monovalent vaccines. Increasing age was associated with increasing odds of receiving boosters (Table 3).

### 3.4. Comparing Primary Complete Vaccination and Boosters between Key HIV Groups and the HIV-Negative Population

**Figure 1:**
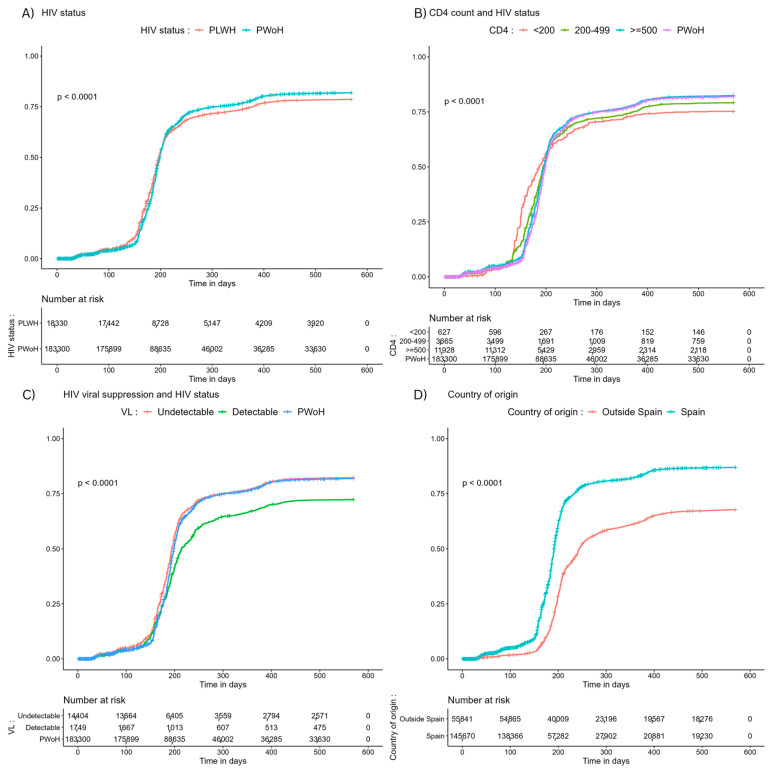
Cumulative incidence of complete primary SARS-CoV-2 vaccination stratified by (**A**) HIV status, (**B**) CD4 count and HIV status, (**C**) HIV viral suppression and HIV status, and (**D**) country of origin and HIV status. Abbreviations: PWH, people with HIV; PWoH, people without HIV; VL, HIV RNA viral load.

Compared to PWoH, individuals living with HIV had higher vaccination rates against SARS-CoV-2 in the first 200 days of the vaccination campaign. However, after 16 months, complete vaccination coverage was significantly lower among PWH (*p* < 0.001). We observed a similar vaccination coverage between PWoH and PWH with CD4 counts >500 cells/μL. However, among PWH with CD4 counts <200 cells/μL, complete primary vaccination coverage was significantly lower (*p* < 0.001). Similarly, primary vaccination coverage was similar between PWoH and PWH with undetectable HIV viral loads but was significantly lower among PWH with detectable viral loads (*p* < 0.001). Significant differences were also observed in primary vaccination coverage between the host Spanish population and individuals of non-Spanish origin (*p* < 0.001) (Figure 1).

**Figure 2:**
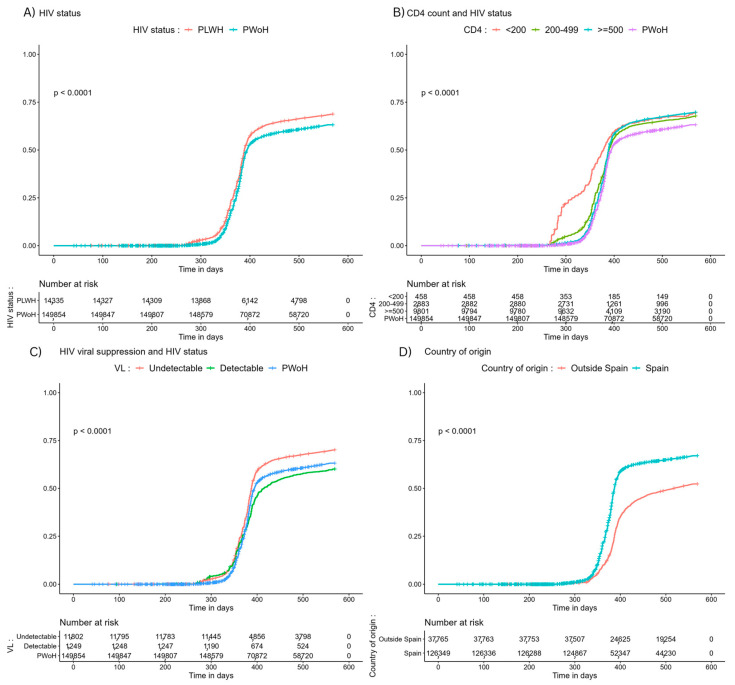
Cumulative incidence of booster SARS-CoV-2 vaccinations stratified by (**A**) HIV status, (**B**) CD4 count and HIV status, (**C**) HIV viral suppression and HIV status, and (**D**) country of origin and HIV status. Abbreviations: PWH, people with HIV; PWoH, people without HIV; VL, HIV RNA viral load.

Regarding booster vaccinations, the coverage was higher among PWH compared to PWoH (*p* < 0.001). PWH with CD4 < 200 cells/μL received more boosters compared to PWoH (*p* < 0.001). Booster reception among PWH with undetectable viral loads; however, remained significantly lower compared to PWoH and PWH with undetectable HIV viral loads. In terms of country of origin, booster reception was lower among individuals of non-Spanish origin (Figure 2). 

## 4. Discussion

PWH may be more susceptible to severe COVID-19 outcomes [4]. Therefore, ensuring equitable access to SARS-CoV-2 vaccines for this vulnerable population is vital [24]. Furthermore, specific sub-populations, such as older individuals, those with lower CD4 cell counts, detectable HIV viremia, and chronic comorbidities, face elevated risks and potentially worse clinical outcomes from HIV/COVID-19 co-infection [6]. Researchers recommend that prevention strategies should target these particular sub-groups [6,25]. 

In our study, the primary vaccination coverage in the overall population (PWH and PWoH) was 81.4%, surpassing the regional average of 75.1% reported by the European Centre for Disease Prevention and Control (ECDC) [26]. Similarly, the observed coverage of booster vaccinations in our cohort was 65.3%, also exceeding the reported regional average of 54.8% [26]. These findings underscore the significant efforts made by the Government of Spain and the Spanish Agency for Medicines and Healthcare Products (AEMPS) to implement nationwide vaccination strategies, particularly emphasizing access for the most vulnerable groups. They also underline historical vaccine acceptance and favourable willingness to be vaccinated in Spain [27]. However, it is worth noting that while our observed primary vaccination rate exceeds the regional average, it falls slightly below the reported 84.9% for the same period in Spain [28]. This suggests that further work is needed to achieve optimal vaccination coverage in the general population of Catalonia.

The observed lower SARS-CoV-2 primary vaccination rates among PWH compared to PWoH in Catalonia are concerning. This trend aligns with a global HIV cohort [17] and a study conducted in New York [29], indicating a consistent pattern of reduced vaccination coverage among PWH. The observed disparities in SARS-CoV-2 vaccination rates among PWH could be attributed to various factors, including potential barriers and hesitancy toward vaccination [18]. Even before the pandemic, vaccine hesitancy was recognized as a significant global health concern by the World Health Organization [30]. Concerns about the safety of the new SARS-CoV-2 vaccines have been a primary reason for vaccine refusal, as highlighted in reports [31]. Furthermore, access barriers, including limited availability or insufficient information tailored to the needs of PWH, could contribute to lower vaccination rates within this population [31]. 

Multiple unmeasured factors, including differences in the nature of jobs, might influence this reduced vaccination rate. The study also identified sociodemographic factors such as migration status and socio-economic deprivation as predictors of lower vaccine uptake, mirroring the findings of a New York-based report [29]. Despite the Catalan Healthcare system offering universal and cost-free access to all citizens, regardless of administrative status, studies in Catalonia have shown a correlation between migrants, socio-economic deprivation, and limited healthcare service utilization [32]. Additionally, non-Spanish origin encompasses a wide range of factors including cultural disparities, language barriers, and specific community beliefs and practices which might significantly influence perceptions and access to vaccination services. Of particular concern is the lower coverage of complete primary vaccination among PWH with CD4 counts below 200 cells/μL and those with detectable HIV viral load, which mirrors findings from previous US studies [17,33]. These individuals are likely to be at higher risk of severe COVID-19 clinical outcomes due to their compromised immune status [6]. The presence of detectable viral loads has been associated with a younger age, a higher likelihood of missing medical appointments, and a lack of treatment adherence [34]. These factors could also partially explain why this important sub-population is under-vaccinated and underscores the necessity for comprehensive, patient-centered approaches to support PWH in achieving optimal health outcomes. Historically, PWH have shown hesitancy toward vaccinations compared to their HIV-negative counterparts, and understanding this reluctance in future studies could be crucial to tailoring effective interventions.

Consistent with an earlier study conducted in Catalonia [35], individuals previously infected with SARS-CoV-2 showed lower vaccine uptake. This can be linked to Catalonia’s vaccination strategy, which delayed schedules for individuals with prior infections for their subsequent vaccination until six months after a confirmed SARS-CoV-2 diagnosis [36], presuming some level of immunity from their past exposure. Evaluating both natural and vaccine-induced immunity is crucial in understanding COVID-19 risk, especially in high-transmission risk settings.

Recommendations for vaccinating PWH favored mRNA vaccines over Ad5 vector SARS-CoV-2 ones [37] due to concerns arising from the Step [38] and Phambili [39] studies, revealing increased HIV-1 acquisition risk in Ad5-vaccinated men. In our study, 75.8% of PWH initially received mRNA vaccines. This could result from using public spaces for vaccinations in the early phase of the pandemic to improve accessibility and coverage without compulsory HIV-status disclosure. However, during booster doses, 99.6% of PWH received mRNA vaccines, likely because booster vaccinations were handled by HIV units and vaccines without adenoviral vectors were prioritized. 

Despite the lower overall primary vaccination coverage, PWH showed higher rates of booster dose uptake compared to PWoH. This finding suggests that PWH and their healthcare providers may proactively seek additional doses to bolster their immune response, particularly following reports indicating inadequate immunogenicity and severe clinical outcomes from HIV/SARS-CoV-2 co-infection. It could also imply reluctance among the general population to receive booster shots, as reported in other settings [40,41]. Studies in the general population have linked this reluctance to perceived or reported side effects from the primary vaccination series, perceived (in)effectiveness of booster doses, low perception of COVID-19 risk, safety concerns, and lower education levels [40,41]. 

The higher odds of booster dose reception among PWH with CD4 levels below 200 cells/μL, aligning with public health recommendations [15], is encouraging due to their increased susceptibility to severe COVID-19 outcomes. However, the lower booster coverage among PWH with detectable viral loads requires attention. PWH with detectable viral loads might face challenges such as non-adherence to antiretrovirals, missing appointments, or engaging in risky behaviors, all of which could predict lower reception of booster doses. During the surge of the Omicron variant in Catalonia, the monovalent SARS-CoV-2 vaccine was introduced. Studies have demonstrated higher immunogenicity of monovalent booster doses against Omicron compared to a two-dose regimen [42,43]. Developing strategies to enhance booster vaccinations in vulnerable populations, like PWH, during this period was pertinent. These individuals stand to benefit greatly from booster doses due to their increased risk of breakthrough infections [44].

Our study has some notable strengths. To our knowledge, this is the first comprehensive evaluation of SARS-CoV-2 booster vaccination coverage among matched people with and without HIV. Additionally, the study used adequate matching of key sociodemographic factors to address potential differences between PWH and PWoH, enhancing the validity. 

The study has some limitations as well. Firstly, the socioeconomic deprivation measure is an ecological variable based on an individual’s place of residence. A person’s place of residence may indeed not necessarily reflect their socioeconomic deprivation. Secondly, we did not report data regarding the post-vaccination experiences of participants, particularly side effects from the primary vaccination doses, which might influence participants’ willingness to receive booster vaccinations. Thirdly, due to the nature of our study design, there might be residual confounding as certain variables, such as religion and occupation, factors that could impact vaccine reception, are not reported in our databases. Additionally, self-made home SARS-CoV-2 antigen test results are not available in our database. We are not able to report if this influenced vaccine reception. 

## 5. Conclusions

In conclusion, the study highlighted concerning discrepancies in SARS-CoV-2 vaccination rates between PWH and those without HIV in Catalonia, Spain. We observed lower primary vaccination rates among PWH compared to PWoH, even though PWH tended to have a higher prevalence of comorbidities. This indicates potential barriers to vaccination access or healthcare linkage among this group, especially for migrants, individuals experiencing socioeconomic deprivation, those with lower CD4 counts, and detectable HIV viral loads. However, the study uncovered a contrasting trend concerning booster vaccinations. PWH, particularly those with lower CD4 counts, were more likely to receive booster doses compared to PWoH. This is a positive finding suggesting increased awareness of booster shots among treating HIV physicians and PWH, particularly those with immunosuppression. Overall, the study underscores the need for targeted interventions to address the disparities in vaccination coverage among vulnerable populations. Improving access to primary vaccinations for PWH, especially those with lower CD4 counts and detectable viral loads, is crucial. Additionally, efforts to ensure equitable access to vaccines among migrants and socioeconomically deprived individuals are imperative. Improving HIV vaccination requires the involvement of HIV physicians, units, and effective communication emphasizing vaccine safety and efficacy.

## Figures and Tables

**Table 1:** Baseline characteristics of study participants according to HIV status.

	Total, n = 201,630	PWH, n = 18,330	PwoH, n = 183,300	*p*-Value
Characteristic	n (%)	n (%)	n (%)	
Sex ^a^				>0.99
Male	165,682 (82.2)	15,062 (82.2)	150,620 (82.2)	
Female	35,948 (17.8)	3268 (17.8)	32,680 (17.8)	
Age category, y ^b^				>0.99
16–30	17,985 (8.9)	1635 (8.9)	16,350 (8.9)	
31–40	48,081 (23.8)	4371 (23.8)	43,710 (23.8)	
41–50	62,909 (31.2)	5719 (31.2)	57,190 (31.2)	
51–60	52,602 (26.1)	4782 (26.1)	47,820 (26.1)	
61–70	15,004 (7.4)	1364 (7.4)	13,640 (7.4)	
>70	5049 (2.5)	459 (2.5)	4590 (2.5)	
Country of origin ^c^				<0.001
Spain	145,670 (72.2)	10,666 (58.2)	135,004 (73.7)	
Outside Spain	55,841 (27.7)	7662 (41.8)	48,179 (26.3)	
Missing	119 (0.1)	2 (0)	117 (0.1)	
Socioeconomic deprivation *				>0.99
Least deprived	99,836 (49.5)	9076 (49.5)	90,760 (49.5)	
Mildly deprived	38,544 (19.1)	3504 (19.1)	35,040 (19.1)	
Moderately/severely deprived	58,652 (29.1)	5332 (29.1)	53,320 (29.1)	
Missing	4598 (2.3)	418 (2.3)	4180 (2.3)	
Number of comorbidities				<0.001
0	87,154 (43.2)	5024 (27.4)	82,130 (44.8)	
1	46,042 (22.8)	4083 (22.3)	41,959 (22.9)	
2	29,102 (14.4)	3299 (18)	25,803 (14.1)	
3	18,348 (9.1)	2387 (13)	15,961 (8.7)	
≥4	20,984 (10.4)	3537 (19.3)	17,447 (9.5)	
Type of comorbidities				
Respiratory disease	18,472 (9.2)	3852 (21)	14,620 (8)	<0.001
Cardiovascular disease	20,974 (10.4)	2925 (16)	18,049 (9.8)	<0.001
Autoimmune disease	17,120 (8.5)	2019 (11)	15,101 (8.2)	<0.001
Chronic kidney disease	11,360 (5.6)	1622 (8.8)	9738 (5.3)	<0.001
Chronic liver disease	6764 (3.4)	3530 (19.3)	3234 (1.8)	<0.001
Neuropsychiatric conditions	59,822 (29.7)	9107 (49.7)	50,715 (27.7)	<0.001
Diabetes (type I and II)	10,949 (5.4)	1043 (5.7)	9906 (5.4)	0.1
Metabolic disease	40,981 (20.3)	4221 (23)	36,760 (20.1)	<0.001
Cancer	9004 (4.5)	1821 (9.9)	7183 (3.9)	<0.001
Hypertension	36,362 (18)	3688 (20.1)	32,674 (17.8)	<0.001
Obesity	28,874 (14.3)	1801 (9.8)	27,073 (14.8)	<0.001
Previous SARS-CoV-2 diagnosis				<0.001
Yes	25,093 (12.4)	2454 (13.4)	22,639 (12.4)	
No	176,537 (87.6)	15,876 (86.6)	160,661 (87.6)	
HIV acquisition risk group				
PWID		2360 (12.9)		
MSM		9761 (53.3)		
Male heterosexual		2443 (13.3)		
Female sexual tranmission		2419 (13.2)		
Other		519 (2.8)		
Missing		828 (4.5)		
Years since HIV diagnosis, median (IQR)		11.57 (5.91–18.57)		
CD4 count (cells/μL) category				
<200		627 (3.4)		
200–499		3665 (20)		
≥500		11,928 (65.1)		
Missing		2110 (11.5)		
CD4 count (cells/μL), median (IQR)		680 (486–908)		
CD4/CD8 ratio, median (IQR)		0.85 (0.57–1.2)		
Plasma HIV-RNA				
Detectable		1749 (9.5)		
Undetectable		14,404 (78.6)		
Missing		2177 (11.9)		
Years on ART, median (IQR) ^d^		8.75 (4.16–14.41)		
On Treatment				
Yes		14,685 (80.1)		
No		3645 (19.9)		

Abbreviations: PWH, people with HIV; PWoH, people without HIV; SARS-CoV-2, severe acute respiratory syndrome coronavirus 2; IQR, interquartile range; PWID, people who inject drugs; MSM, men who have sex with men; ART, antiretroviral therapy. ^a^ Sex as assigned birth. ^b^ Age for all patients was as of 1 January 2021. ^c^ Country of origin was as indicated by the Public Data Analysis for Health Research and Innovation Program of Catalonia (PADRIS), recorded as Spanish or Non-Spanish. ^d^ Years on ART was defined as the difference in time between the first treatment administration date to the latest treatment date or the latest hospital visit if the last treatment date is missing. * Socioeconomic deprivation is based on the index of the Catalan government according to the basic health area (ABS) of residence. This index is based on five indicators which are: proportion of manual workers, proportion of residents with low education level, proportion with low income, rate of premature mortality, and rate of avoidable hospitalization.

**Table 2:** SARS-CoV-2 vaccination coverage between people with and without HIV.

	Total	PWH	PWoH	*p*-Value
Primary vaccination	n (%)	n (%)	n (%)	<0.001
Unvaccinated	29,606 (14.7)	3343 (18.2)	26,263 (14.3)	
Incomplete	7835 (3.9)	652 (3.6)	7183 (3.9)	
Complete	164,189 (81.4)	14,335 (78.2)	149,854 (81.8)	
Primary vaccination type				<0.001
BNT162	105,743 (61.5)	7924 (52.9)	97,819 (62.3)	
ChAdOx1-S	16,668 (9.7)	1436 (9.6)	15,232 (9.7)	
mRNA-1273	29,505 (17.2)	3737 (24.9)	25,768 (16.4)	
Ad26.COV2.S	13,346 (7.8)	1321 (8.8)	12,025 (7.7)	
Combined	6762 (3.9)	569 (3.8)	6193 (3.9)	
Booster doses				<0.001
Yes	104,332 (63.5)	9823 (68.5)	94,509 (63.1)	
No	59,857 (36.5)	4512 (31.5)	55,345 (36.9)	
Booster doses type				<0.001
BNT162	13,973 (13.4)	1413 (14.4)	12,560 (13.3)	
ChAdOx1-S	26 (0)	4 (0)	22 (0)	
mRNA-1273	90,250 (86.5)	8372 (85.2)	81,878 (86.6)	
Ad26.COV2.S	10 (0)	2 (0)	8 (0)	
Combined	40 (0)	17 (0.2)	23 (0)	
Other	33 (0)	15 (0.2)	18 (0)	
Median time between primary and booster dose, months (IQR)	6.44 (5.98–7.1)	6.44 (5.92–7.13)	6.44 (6.02–7.1)	<0.001

Abbreviations: PWH, people with HIV; PWoH, people without HIV; IQR, interquaartile range.

**Table 3:** Factors associated with (a) complete and (b) booster vaccine reception in logistic regression analysis.

	Complete Primary Vaccination		Booster Vaccination	
	aOR (95% CI)	*p*-Value	aOR (95% CI)	*p*-Value
**HIV Status**				
Negative	1 (ref)		1 (ref)	
Positive	0.86 (0.82, 0.89)	<0.001	1.41 (1.36, 1.47)	<0.001
**Sex**				
Male	1 (ref)		1 (ref)	
Female	1.1 (1.07, 1.14)	<0.001	1.03 (1, 1.06)	0.091
**Age category, y**				
16–30	1 (ref)		1 (ref)	
31–40	1.3 (1.25, 1.36)	<0.001	1.61 (1.54, 1.68)	<0.001
41–50	1.79 (1.72, 1.87)	<0.001	2.76 (2.65, 2.88)	<0.001
51–60	2.21 (2.11, 2.31)	<0.001	4.67 (4.46, 4.89)	<0.001
61–70	2.12 (1.99, 2.27)	<0.001	9.88 (9.26, 10.55)	<0.001
>70	2.73 (2.42, 3.07)	<0.001	17.48 (15.53, 19.67)	<0.001
**Place of Birth**				
Spain	1 (ref)		1 (ref)	
Outside Spain	0.39 (0.38, 0.4)	<0.001	0.75 (0.73, 0.77)	<0.001
Missing				
Socioeconomic deprivation *				
Least deprived	1 (ref)		1 (ref)	
Mildly deprived	0.87 (0.84, 0.9)	<0.001	0.8 (0.78, 0.83)	<0.001
Moderately/severely deprived	0.87 (0.85, 0.9)	<0.001	0.77 (0.75, 0.79)	<0.001
**Number of comorbidities**				
0	1 (ref)		1 (ref)	
1	1.26 (1.22, 1.3)	<0.001	1.01 (0.98, 1.04)	0.599
2	1.45 (1.39, 1.51)	<0.001	1.07 (1.04, 1.11)	<0.001
3	1.64 (1.56, 1.73)	<0.001	1.13 (1.08, 1.18)	<0.001
≥4	1.78 (1.69, 1.88)	<0.001	1.27 (1.21, 1.32)	<0.001
**Previous SARS-CoV-2 diagnosis**				
No	1 (ref)		1 (ref)	
Yes	0.2 (0.19, 0.2)	<0.001	0.24 (0.23, 0.25)	<0.001

Abbreviations: OR, odds ratio; aOR, adjusted odds ratio; SARS-CoV-2, severe acute respiratory syndrome coronavirus 2. Models adjusted for age, sex, country of origin, socioeconomic deprivation, prior SARS-CoV-2 diagnosis, number of comorbidities, and HIV status. * Socioeconomic deprivation is based on the index of the Catalan government according to the basic health area (ABS) of residence. This index is based on five indicators which are: proportion of manual workers, proportion of residents with low education level, proportion with low income, rate of premature mortality, and rate of avoidable hospitalization.

## Data Availability

The study protocol and statical code are available from the corresponding author upon request. The data for this study accessed on 1 October 2022 are available at the Centre for Epidemiological Studies of Sexually Transmitted Diseases and HIV/AIDS in Catalonia (CEEISCAT), the coordinating centre of the PISCIS cohort, the PADRIS, and from each of the collaborating hospitals upon request via https://pisciscohort.org/contacte/.

## Supplementary Materials

The following supporting information can be downloaded at: https://www.mdpi.com/article/10.3390/vaccines12010044/s1, Table S1: Factors associated with complete vaccine reception among HIV-negative participants in logistic regression analysis; Table S2: Factors associated with booster vaccine reception among HIV-negative participants in logistic regression analysis; Table S3: Factors associated with complete vaccine reception among PWH in logistic regression analysis; Table S4: Factors associated with booster vaccine reception among PWH in logistic regression analysis.

## Appendix A

Eleven groups of the most prevalent chronic comorbidities among people living with and without HIV in Catalonia with corresponding International Classification of Diseases, Ninth and Tenth Revision, Clinical Modification (ICD-9/10-CM) codes.

**Table:**

Disease Groups	ICD-10-CM	Description (ICD-10)	ICD-9-CM	Description (ICD-9)
AUTOIMMUNE	I731	Thromboangiitis obliterans [Buerger]	443.1	Obliterating thromboangeitis (Buerger’s disease)
AUTOIMMUNE	L10	Pemphigus		
AUTOIMMUNE	L12	Pemphigoid		
AUTOIMMUNE	L40	Psoriasis		
AUTOIMMUNE	L41	Parapsoriasis		
AUTOIMMUNE	L93	Lupus erythematosus		
AUTOIMMUNE	L94	Other localized connective tissue disorders		
AUTOIMMUNE	L95	Vasculitis limited to skin, not elsewhere classified		
AUTOIMMUNE	M30	Polyarteritis nodosa and related conditions		
AUTOIMMUNE	M31	Other necrotizing vasculopathies		
AUTOIMMUNE	M32	Systemic lupus erythematosus		
AUTOIMMUNE	M33	Dermatopolymyositis		
AUTOIMMUNE	M34	Systemic sclerosis		
AUTOIMMUNE	M35	Other systemic involvement of connective tissue		
AUTOIMMUNE	M36	Systemic disorders of connective tissue in diseases classified elsewhere		
AUTOIMMUNE	M023	Reiter disease		
AUTOIMMUNE	M05	Seropositive rheumatoid arthritis		
AUTOIMMUNE	M06	Other rheumatoid arthritis		
AUTOIMMUNE	M07	Psoriatic and enteropathic arthropathies		
AUTOIMMUNE	M08	Juvenile arthritis		
AUTOIMMUNE	M09	Juvenile arthritis in diseases classified elsewhere		
AUTOIMMUNE	M10	Gout		
AUTOIMMUNE	M11	Other crystal arthropathies		
AUTOIMMUNE	M12	Other specific arthropathies		
AUTOIMMUNE	M13	Other arthritis		
AUTOIMMUNE	M14	Arthropathies in other diseases classified elsewhere		
AUTOIMMUNE	M45	Ankylosing spondylitis		
AUTOIMMUNE	M460	Spinal enthesopathy		
AUTOIMMUNE	M461	Sacroiliitis, not elsewhere classified	720.2	Sacroiliitis, not elsewhere classified
AUTOIMMUNE	M468	Other specified inflammatory spondylopathies		
AUTOIMMUNE	M469	Inflammatory spondylopathy, unspecified		
AUTOIMMUNE	K50	Crohn disease [regional enteritis]		
AUTOIMMUNE	K51	Ulcerative colitis		
AUTOIMMUNE	G35	Multiple sclerosis	340	Multiple sclerosis
CANCER	C81	Hodgkin lymphoma		
CANCER	C82	Follicular lymphoma		
CANCER	C83	Non-follicular lymphoma		
CANCER	C84	Mature T/NK-cell lymphomas		
CANCER	C85	Other and unspecified types of non-Hodgkin lymphoma		
CANCER	C86	Other specified types of T/NK-cell lymphoma		
CANCER	C88	Malignant immunoproliferative diseases		
CANCER	C90	Multiple myeloma and malignant plasma cell neoplasms		
CANCER	C91	Lymphoid leukaemia		
CANCER	C92	Myeloid leukaemia		
CANCER	C93	Monocytic leukaemia		
CANCER	C94	Other leukaemias of specified cell type		
CANCER	C95	Leukaemia of unspecified cell type		
CANCER	C96	Other and unspecified malignant neoplasms of lymphoid, haematopoietic and related tissue		
CANCER	C	Malignant neoplasms		
CANCER	D00	Carcinoma in situ of oral cavity, oesophagus and stomach		
CANCER	D01	Carcinoma in situ of other and unspecified digestive organs		
CANCER	D02	Carcinoma in situ of middle ear and respiratory system		
CANCER	D03	Melanoma in situ		
CANCER	D04	Carcinoma in situ of skin		
CANCER	D05	Carcinoma in situ of breast		
CANCER	D06	Carcinoma in situ of cervix uteri		
CANCER	D07	Carcinoma in situ of other and unspecified genital organs		
CANCER	D09	Carcinoma in situ of other and unspecified sites		
CANCER	D320	Benign neoplasm: Cerebral meninges	225.2	Benign neoplasm of cerebral meninges
CANCER	D321	Benign neoplasm: Spinal meninges	225.4	Benign neoplasm of spinal meninges
CANCER	D329	Benign neoplasm: Meninges, unspecified		
CANCER	D330	Benign neoplasm: Brain, supratentorial	225.0	Benign neoplasm of brain and other parts of nervous system
CANCER	D331	Benign neoplasm: Brain, infratentorial		
CANCER	D332	Benign neoplasm: Brain, unspecified		
CANCER	D333	Benign neoplasm: Cranial nerves	225.1	Benign neoplasm of cranial nerves convert
CANCER	D334	Benign neoplasm: Spinal cord	225.3	Benign neoplasm of spinal cord
CANCER	Q85	Phakomatoses, not elsewhere classified		
CANCER	C81	Hodgkin lymphoma		
CANCER	C82	Follicular lymphoma		
CANCER	C83	Non-follicular lymphoma		
CANCER	C84	Mature T/NK-cell lymphomas		
CANCER	C85	Other and unspecified types of non-Hodgkin lymphoma		
CANCER	C86	Other specified types of T/NK-cell lymphoma		
CANCER	C88	Malignant immunoproliferative diseases		
CANCER	C90	Multiple myeloma and malignant plasma cell neoplasms		
CANCER	C91	Lymphoid leukaemia		
CANCER	C92	Myeloid leukaemia		
CANCER	C93	Monocytic leukaemia		
CANCER	C94	Other leukaemias of specified cell type		
CANCER	C95	Leukaemia of unspecified cell type		
CANCER	C96	Other and unspecified malignant neoplasms of lymphoid, haematopoietic and related tissue		
CARDIOVASCULAR	I48	Atrial fibrillation and flutter		
CARDIOVASCULAR	I441	Atrioventricular block, second degree	426.12	Mobitz (type) II atrioventricular block
CARDIOVASCULAR	I441	Atrioventricular block, second degree	426.13	Other second degree atrioventricular block
CARDIOVASCULAR	I442	Atrioventricular block, complete	426.0	Atrioventricular block, complete
CARDIOVASCULAR	I443	Other and unspecified atrioventricular block		
CARDIOVASCULAR	I453	Trifascicular block	426.54	Trifascicular block
CARDIOVASCULAR	I455	Other specified heart block	426.6	Other heart block
CARDIOVASCULAR	Z950	Presence of cardiac pacemaker	V45.01	Status cardiac pacemaker
CARDIOVASCULAR	I05	Rheumatic mitral valve diseases		
CARDIOVASCULAR	I06	Rheumatic aortic valve diseases		
CARDIOVASCULAR	I07	Rheumatic tricuspid valve diseases		
CARDIOVASCULAR	I08	Multiple valve diseases		
CARDIOVASCULAR	I091	Rheumatic diseases of endocardium, valve unspecified	397.9	Rheumatic diseases of endocardium, valve unspecified.
CARDIOVASCULAR	I098	Other specified rheumatic heart diseases		
CARDIOVASCULAR	I34	Nonrheumatic mitral valve disorders		
CARDIOVASCULAR	I35	Nonrheumatic aortic valve disorders		
CARDIOVASCULAR	I36	Nonrheumatic tricuspid valve disorders		
CARDIOVASCULAR	I37	Pulmonary valve disorders		
CARDIOVASCULAR	I38	Endocarditis, valve unspecified	424.90	Endocarditis, valve unspecified, unspecified cause.
CARDIOVASCULAR	I38	Endocarditis, valve unspecified	424.99	Endocarditis, valve unspecified.
CARDIOVASCULAR	I390	Mitral valve disorders in diseases classified elsewhere		
CARDIOVASCULAR	I391	Aortic valve disorders in diseases classified elsewhere		
CARDIOVASCULAR	I392	Tricuspid valve disorders in diseases classified elsewhere		
CARDIOVASCULAR	I393	Pulmonary valve disorders in diseases classified elsewhere		
CARDIOVASCULAR	I394	Multiple valve disorders in diseases classified elsewhere		
CARDIOVASCULAR	Q22	Congenital malformations of pulmonary and tricuspid valves		
CARDIOVASCULAR	Q23	Congenital malformations of aortic and mitral valves		
CARDIOVASCULAR	Z952	Presence of prosthetic heart valve	V43.3	Heart valve replaced by other means
CARDIOVASCULAR	Z953	Presence of xenogenic heart valve	V42.2	Heart valve replaced by transplant
CARDIOVASCULAR	Z954	Presence of other heart-valve replacement	V42.2	
CARDIOVASCULAR	G45	Transient cerebral ischaemic attacks and related syndromes		
CARDIOVASCULAR	G46	Vascular syndromes of brain in cerebrovascular diseases		
CARDIOVASCULAR	I60	Subarachnoid haemorrhage		
CARDIOVASCULAR	I61	Intracerebral haemorrhage		
CARDIOVASCULAR	I62	Other nontraumatic intracranial haemorrhage		
CARDIOVASCULAR	I63	Cerebral infarction		
CARDIOVASCULAR	I64	Stroke, not specified as haemorrhage or infarction		
CARDIOVASCULAR	I67	Other cerebrovascular diseases		
CARDIOVASCULAR	I69	Sequelae of cerebrovascular disease		
CARDIOVASCULAR	I110	Hypertensive heart disease with (congestive) heart failure	402.01	Malignant hypertensive heart disease with heart failure
CARDIOVASCULAR	I110	Hypertensive heart disease with (congestive) heart failure	402.11	Malignant hypertensive heart disease with heart failure
CARDIOVASCULAR	I110	Hypertensive heart disease with (congestive) heart failure	402.91	Unspecified hypertensive heart disease
CARDIOVASCULAR	I130	Hypertensive heart and renal disease with (congestive) heart failure	404.01	Hypertensive heart and renal disease, with congestive heart failure, malignant
CARDIOVASCULAR	I130	Hypertensive heart and renal disease with (congestive) heart failure	404.11	Hypertensive heart and chronic kidney disease with heart failure and stage 1 through stage 4 chronic kidney disease.
CARDIOVASCULAR	I130	Hypertensive heart and renal disease with (congestive) heart failure	404.91	Hypertensive heart and chronic kidney disease with heart failure and stage 1 through stage 4 chronic kidney disease
CARDIOVASCULAR	I132	Hypertensive heart and renal disease with both (congestive) heart failure and renal failure	404.03	Hypertensive HF and CKD- Kidney Failure
CARDIOVASCULAR	I132	Hypertensive heart and renal disease with both (congestive) heart failure and renal failure	404.13	Hypertensive heart and chronic kidney disease with heart failure and with stage 5 chronic kidney disease
CARDIOVASCULAR	I132	Hypertensive heart and renal disease with both (congestive) heart failure and renal failure	404.93	Unspecified w/chf and renal failure
CARDIOVASCULAR	I27	Other pulmonary heart diseases		
CARDIOVASCULAR	I280	Arteriovenous fistula of pulmonary vessels	417.0	Arteriovenous fistula of pulmonary vessels
CARDIOVASCULAR	I42	Cardiomyopathy		
CARDIOVASCULAR	I43	Cardiomyopathy in diseases classified elsewhere	425.8	Cardiomyopathy in other diseases classified elsewhere
CARDIOVASCULAR	I50	Heart failure		
CARDIOVASCULAR	I515	Myocardial degeneration	429.1	Myocardial degeneration
CARDIOVASCULAR	I517	Cardiomegaly	429.3	Cardiomegaly
CARDIOVASCULAR	I528	Other heart disorders in other diseases classified elsewhere		
CARDIOVASCULAR	Z941	Heart transplant status	V42.1	Heart transplant status
CARDIOVASCULAR	Z943	Heart and lungs transplant status	V42.1	
CARDIOVASCULAR	Z943	Heart and lungs transplant status	V42.6	Lung transplant status
CARDIOVASCULAR	I20	Angina pectoris		
CARDIOVASCULAR	I21	Acute myocardial infarction		
CARDIOVASCULAR	I22	Subsequent myocardial infarction		
CARDIOVASCULAR	I24	Other acute ischaemic heart diseases		
CARDIOVASCULAR	I25	Chronic ischaemic heart disease		
CARDIOVASCULAR	Z951	Presence of aortocoronary bypass graft	V45.81	Aortocoronary bypass status
CARDIOVASCULAR	Z955	Presence of coronary angioplasty implant and graft	V45.82	Ercutaneous transluminal coronary angioplasty status
CARDIOVASCULAR	I09	Other rheumatic heart diseases		
CARDIOVASCULAR	I281	Aneurysm of pulmonary artery	417.1	Aneurysm of pulmonary artery
CARDIOVASCULAR	I310	Chronic adhesive pericarditis	423.1	Adhesive pericarditis
CARDIOVASCULAR	I311	Chronic constrictive pericarditis	423.2	Constrictive pericarditis.
CARDIOVASCULAR	I456	Pre-excitation syndrome	426.7	Anomalous atrioventricular excitation.
CARDIOVASCULAR	I456	Pre-excitation syndrome	426.81	Lown-Ganong-Levine syndrome.
CARDIOVASCULAR	I495	Sick sinus syndrome	427.81	Sinoatrial node dysfunction
CARDIOVASCULAR	I498	Other specified cardiac arrhythmias	427.89	Other specified cardiac dysrhythmias
CARDIOVASCULAR	I70	Atherosclerosis		
CARDIOVASCULAR	I71	Aortic aneurysm and dissection		
CARDIOVASCULAR	I72	Other aneurysm and dissection		
CARDIOVASCULAR	I790	Aneurysm of aorta in diseases classified elsewhere	441.9	Aortic aneurysm of unspecified site without mention of rupture
CARDIOVASCULAR	I791	Aortitis in diseases classified elsewhere	443.81	Peripheral angiopathy in diseases classified elsewhere
CARDIOVASCULAR	I950	Idiopathic hypotension	458.1	Chronic hypotension
CARDIOVASCULAR	I951	Orthostatic hypotension	458.0	Orthostatic hypotension
CARDIOVASCULAR	I958	Other hypotension		
CARDIOVASCULAR	Q20	Congenital malformations of cardiac chambers and connections		
CARDIOVASCULAR	Q21	Congenital malformations of cardiac septa		
CARDIOVASCULAR	Q24	Other congenital malformations of heart		
CARDIOVASCULAR	Q25	Congenital malformations of great arteries		
CARDIOVASCULAR	Q26	Congenital malformations of great veins		
CARDIOVASCULAR	Q27	Other congenital malformations of peripheral vascular system		
CARDIOVASCULAR	Q28	Other congenital malformations of circulatory system		
CARDIOVASCULAR	Z958	Presence of other cardiac and vascular implants and grafts		
CARDIOVASCULAR	Z959	Presence of cardiac and vascular implant and graft, unspecified	V45.00	Cardiac device in situ
CARDIOVASCULAR	I091	Rheumatic diseases of endocardium, valve unspecified	397.9	Rheumatic diseases of endocardium, valve unspecified
CARDIOVASCULAR	I098	Other specified rheumatic heart diseases		
CARDIOVASCULAR	I702	Atherosclerosis of arteries of extremities		
CARDIOVASCULAR	I780	Hereditary haemorrhagic telangiectasia	448.0	Hereditary hemorrhagic telangiectasia.
CARDIOVASCULAR	I83	Varicose veins of lower extremities		
CARDIOVASCULAR	I87	Other disorders of veins		
CARDIOVASCULAR	I89	Other noninfective disorders of lymphatic vessels and lymph nodes		
CARDIOVASCULAR	I972	Postmastectomy lymphoedema syndrome	457.0	Postmastectomy lymphedema syndrome
CARDIOVASCULAR	Q820	Hereditary lymphoedema	757.0	Hereditary edema of legs
HYPERTENSION	I10	Essential (primary) hypertension	401.0	Malignant essential hypertension
HYPERTENSION	I10	Essential (primary) hypertension	401.1	Benign essential hypertension
HYPERTENSION	I10	Essential (primary) hypertension	401.9	Unspecified essential hypertension
HYPERTENSION	I11	Hypertensive heart disease		
HYPERTENSION	I12	Hypertensive renal disease		
HYPERTENSION	I13	Hypertensive heart and renal disease		
HYPERTENSION	I15	Secondary hypertension		
CHRONIC KIDNEY DISEASES	I120	Hypertensive renal disease with renal failure	403.01	Hypertensive chronic kidney disease, malignant, with chronic kidney disease stage V or end stage renal disease
CHRONIC KIDNEY DISEASES	I120	Hypertensive renal disease with renal failure	403.11	Hypertensive chronic kidney disease, benign, with chronic kidney disease stage v or end stage renal disease
CHRONIC KIDNEY DISEASES	I120	Hypertensive renal disease with renal failure	403.91	Hypertensive chronic kidney disease, unspecified, with chronic kidney disease stage V or end stage renal disease
CHRONIC KIDNEY DISEASES	I130	Hypertensive heart and renal disease with (congestive) heart failure	404.01	Hypertensive heart and renal disease, with congestive heart failure, malignant
CHRONIC KIDNEY DISEASES	I130	Hypertensive heart and renal disease with (congestive) heart failure	404.11	Hypertensive heart and chronic kidney disease with heart failure and stage 1 through stage 4 chronic kidney disease, or unspecified chronic kidney disease
CHRONIC KIDNEY DISEASES	I130	Hypertensive heart and renal disease with (congestive) heart failure	404.91	Hypertensive heart and chronic kidney disease with heart failure and stage 1 through stage 4 chronic kidney disease, or unspecified chronic kidney disease
CHRONIC KIDNEY DISEASES	I131	Hypertensive heart and renal disease with renal failure		
CHRONIC KIDNEY DISEASES	I132	Hypertensive heart and renal disease with both (congestive) heart failure and renal failure	404.03	Hypertensive heart and chronic kidney disease with heart failure and stage 1 through stage 4 chronic kidney disease, or unspecified chronic kidney disease
CHRONIC KIDNEY DISEASES	I132	Hypertensive heart and renal disease with both (congestive) heart failure and renal failure	404.13	Hypertensive HF and CKD–Kidney Failure
CHRONIC KIDNEY DISEASES	I132	Hypertensive heart and renal disease with both (congestive) heart failure and renal failure	404.93	Hypertensive HF and CKD–Kidney Failure
CHRONIC KIDNEY DISEASES	I139	Hypertensive heart and renal disease, unspecified		
CHRONIC KIDNEY DISEASES	N01	Rapidly progressive nephritic syndrome		
CHRONIC KIDNEY DISEASES	N03	Chronic nephritic syndrome		
CHRONIC KIDNEY DISEASES	N04	Nephrotic syndrome		
CHRONIC KIDNEY DISEASES	N05	Unspecified nephritic syndrome		
CHRONIC KIDNEY DISEASES	N07	Hereditary nephropathy, not elsewhere classified		
CHRONIC KIDNEY DISEASES	N08	Glomerular disorders in diseases classified elsewhere	583.81	Nephritis and nephropathy, not specified as acute or chronic, in diseases classified elsewhere
CHRONIC KIDNEY DISEASES	N11	Chronic tubulo-interstitial nephritis		
CHRONIC KIDNEY DISEASES	N183	Chronic kidney disease, stage 3	585.3	Chronic kidney disease, stage 3 (moderate)
CHRONIC KIDNEY DISEASES	N184	Chronic kidney disease, stage 4	585.4	Chronic kidney disease, Stage IV (severe)
CHRONIC KIDNEY DISEASES	N185	Chronic kidney disease, stage 5	585.5	Chronic kidney disease, stage v
CHRONIC KIDNEY DISEASES	N189	Chronic kidney disease, unspecified	585.9	Chronic kidney disease, unspecified
CHRONIC KIDNEY DISEASES	Q60	Renal agenesis and other reduction defects of kidney		
CHRONIC KIDNEY DISEASES	Q611	Polycystic kidney, autosomal recessive		
CHRONIC KIDNEY DISEASES	Q612	Polycystic kidney, autosomal dominant	753.13	Polycystic kidney, autosomal dominant
CHRONIC KIDNEY DISEASES	Q613	Polycystic kidney, unspecified	753.12	Polycystic kidney, unspecified type
CHRONIC KIDNEY DISEASES	Q614	Renal dysplasia	753.15	Renal dysplasia
CHRONIC KIDNEY DISEASES	Q615	Medullary cystic kidney	753.16	Medullary cystic kidney
CHRONIC KIDNEY DISEASES	Q615	Medullary cystic kidney	753.17	Medullary sponge kidney
CHRONIC KIDNEY DISEASES	Q618	Other cystic kidney diseases	753.19	Other specified cystic kidney disease
CHRONIC KIDNEY DISEASES	Q619	Cystic kidney disease, unspecified	753.10	Cystic kidney disease, unspecified
CHRONIC KIDNEY DISEASES	Z905	Acquired absence of kidney	V45.73	Acquired absence kidney
CHRONIC KIDNEY DISEASES	Z940	Kidney transplant status	V42.0	Organ or tissue replaced by transplant
CHRONIC LIVER DISEASES	B18	Chronic viral hepatitis		
CHRONIC LIVER DISEASES	K70	Alcoholic liver disease		
CHRONIC LIVER DISEASES	K713	Toxic liver disease with chronic persistent hepatitis	573.3	Hepatitis, unspecified
CHRONIC LIVER DISEASES	K714	Toxic liver disease with chronic lobular hepatitis	573.3	Hepatitis, unspecified
CHRONIC LIVER DISEASES	K715	Toxic liver disease with chronic active hepatitis		
CHRONIC LIVER DISEASES	K717	Toxic liver disease with fibrosis and cirrhosis of liver	573.3	Hepatitis, unspecified
CHRONIC LIVER DISEASES	K721	Chronic hepatic failure		
CHRONIC LIVER DISEASES	K73	Chronic hepatitis, not elsewhere classified		
CHRONIC LIVER DISEASES	K74	Fibrosis and cirrhosis of liver		
CHRONIC LIVER DISEASES	K753	Granulomatous hepatitis, not elsewhere classified	573.3	Hepatitis, unspecified
CHRONIC LIVER DISEASES	K754	Autoimmune hepatitis	571.42	Chronic persistent hepatitis
CHRONIC LIVER DISEASES	K758	Other specified inflammatory liver diseases		
CHRONIC LIVER DISEASES	K761	Chronic passive congestion of liver	573.0	Chronic passive congestion of liver
CHRONIC LIVER DISEASES	K761	Chronic passive congestion of liver	573.8	Other disorders of liver
CHRONIC LIVER DISEASES	K766	Portal hypertension	572.3	Portal hypertension
CHRONIC LIVER DISEASES	K767	Hepatorenal syndrome	572.4	Hepatorenal syndrome
CHRONIC LIVER DISEASES	K778	Liver disorders in other diseases classified elsewhere		
CHRONIC LIVER DISEASES	Q446	Cystic disease of liver	751.62	Congenital cystic disease of liver
CHRONIC LIVER DISEASES	Z944	Liver transplant status	V42.7	Liver transplant status
CHRONIC LIVER DISEASES	K700	Alcoholic fatty liver	571.0	Alcoholic fatty liver
CHRONIC LIVER DISEASES	K701	Alcoholic hepatitis		
METABOLIC	E78	Disorders of lipoprotein metabolism and other lipidaemias		
METABOLIC	E20	Hypoparathyroidism		
METABOLIC	E21	Hyperparathyroidism and other disorders of parathyroid gland		
METABOLIC	E22	Hyperfunction of pituitary gland		
METABOLIC	E23	Hypofunction and other disorders of pituitary gland		
METABOLIC	E24	Cushing syndrome		
METABOLIC	E25	Adrenogenital disorders		
METABOLIC	E26	Hyperaldosteronism		
METABOLIC	E27	Other disorders of adrenal gland		
METABOLIC	E28	Ovarian dysfunction		
METABOLIC	E29	Testicular dysfunction		
METABOLIC	E31	Polyglandular dysfunction		
METABOLIC	E34	Other endocrine disorders		
METABOLIC	E35	Disorders of endocrine glands in diseases classified elsewhere	246.8	Other specified disorders of thyroid
METABOLIC	E35	Disorders of endocrine glands in diseases classified elsewhere	255.8	Other specified disorders of adrenal glands
METABOLIC	E35	Disorders of endocrine glands in diseases classified elsewhere	259.8	Other specified endocrine disorders
METABOLIC	E40	Kwashiorkor	260	Kwashiorkor
METABOLIC	E41	Nutritional marasmus	261	Nutritional marasmus
METABOLIC	E42	Marasmic kwashiorkor	260	Kwashiorkor
METABOLIC	E43	Unspecified severe protein-energy malnutrition	262	Other severe protein-calorie malnutrition
METABOLIC	E44	Protein-energy malnutrition of moderate and mild degree		
METABOLIC	E45	Retarded development following protein-energy malnutrition	263.2	Arrested development following protein-calorie malnutrition
METABOLIC	E46	Unspecified protein-energy malnutrition	263.8	Unspecified protein-calorie malnutrition
METABOLIC	E46	Unspecified protein-energy malnutrition	263.9	Unspecified protein-calorie malnutrition
METABOLIC	E64	Sequelae of malnutrition and other nutritional deficiencies		
METABOLIC	E70	Disorders of aromatic amino-acid metabolism		
METABOLIC	E71	Disorders of branched-chain amino-acid metabolism and fatty-acid metabolism		
METABOLIC	E72	Other disorders of amino-acid metabolism		
METABOLIC	E74	Other disorders of carbohydrate metabolism		
METABOLIC	E75	Disorders of sphingolipid metabolism and other lipid storage disorders		
METABOLIC	E76	Disorders of glycosaminoglycan metabolism		
METABOLIC	E77	Disorders of glycoprotein metabolism		
METABOLIC	E79	Disorders of purine and pyrimidine metabolism		
METABOLIC	E80	Disorders of porphyrin and bilirubin metabolism		
METABOLIC	E83	Disorders of mineral metabolism		
METABOLIC	E84	Cystic fibrosis		
METABOLIC	E85	Amyloidosis		
METABOLIC	E88	Other metabolic disorders		
METABOLIC	E89	Postprocedural endocrine and metabolic disorders, not elsewhere classified		
METABOLIC	E891	Postprocedural hypoinsulinaemia	251.3	Postsurgical hypoinsulinemia
METABOLIC	K903	Pancreatic steatorrhoea	579.4	Pancreatic steatorrhea
METABOLIC	K904	Malabsorption due to intolerance, not elsewhere classified		
METABOLIC	K908	Other intestinal malabsorption		
METABOLIC	K909	Intestinal malabsorption, unspecified	579.9	Unspecified intestinal malabsorption
METABOLIC	K912	Postsurgical malabsorption, not elsewhere classified	579.3	Postsurgical malabsorption, not elsewhere classified
METABOLIC	M83	Adult osteomalacia		
METABOLIC	M88	Paget disease of bone [osteitis deformans]		
METABOLIC	N25	Disorders resulting from impaired renal tubular function		
DIABETES	E10	Insulin-dependent diabetes mellitus	250.XX	Diabetes
DIABETES	E11	Non-insulin-dependent diabetes mellitus		
DIABETES	E13	Other specified diabetes mellitus		
DIABETES	E14	Unspecified diabetes mellitus		
OBERSITY	E66	Obesity		
NEUROPSYCHIATRIC	F00	Dementia in Alzheimer disease		
NEUROPSYCHIATRIC	F01	Vascular dementia		
NEUROPSYCHIATRIC	F02	Dementia in other diseases classified elsewhere		
NEUROPSYCHIATRIC	F03	Unspecified dementia		
NEUROPSYCHIATRIC	F051	Delirium superimposed on dementia		
NEUROPSYCHIATRIC	G30	Alzheimer disease		
NEUROPSYCHIATRIC	G31	Other degenerative diseases of nervous system, not elsewhere classified		
NEUROPSYCHIATRIC	F30	Manic episode		
NEUROPSYCHIATRIC	F31	Bipolar affective disorder		
NEUROPSYCHIATRIC	F32	Depressive episode		
NEUROPSYCHIATRIC	F33	Recurrent depressive disorder		
NEUROPSYCHIATRIC	F34	Persistent mood [affective] disorders		
NEUROPSYCHIATRIC	F38	Other mood [affective] disorders		
NEUROPSYCHIATRIC	F39	Unspecified mood [affective] disorder	296.90	Unspecified episodic mood disorder
NEUROPSYCHIATRIC	F412	Mixed anxiety and depressive disorder		
NEUROPSYCHIATRIC	G40	Epilepsy		
NEUROPSYCHIATRIC	B900	Sequelae of central nervous system tuberculosis	137.1	Late effects of central nervous system tuberculosis
NEUROPSYCHIATRIC	D482	Neoplasm of uncertain or unknown behaviour: Peripheral nerves and autonomic nervous system	238.1	Neoplasm of uncertain behavior of connective and other soft tissue
NEUROPSYCHIATRIC	G041	Tropical spastic paraplegia	344.1	Paraplegia
NEUROPSYCHIATRIC	G09	Sequelae of inflammatory diseases of central nervous system	326	Late effects of intracranial abscess or pyogenic infection
NEUROPSYCHIATRIC	G10	Huntington disease	333.4	Huntington’s chorea
NEUROPSYCHIATRIC	G11	Hereditary ataxia		
NEUROPSYCHIATRIC	G12	Spinal muscular atrophy and related syndromes		
NEUROPSYCHIATRIC	G13	Systemic atrophies primarily affecting central nervous system in diseases classified elsewhere		
NEUROPSYCHIATRIC	G24	Dystonia		
NEUROPSYCHIATRIC	G25	Other extrapyramidal and movement disorders		
NEUROPSYCHIATRIC	G26	Extrapyramidal and movement disorders in diseases classified elsewhere	333.99	Other extrapyramidal diseases and abnormal movement disorders
NEUROPSYCHIATRIC	G32	Other degenerative disorders of nervous system in diseases classified elsewhere		
NEUROPSYCHIATRIC	G37	Other demyelinating diseases of central nervous system		
NEUROPSYCHIATRIC	G51	Facial nerve disorders		
NEUROPSYCHIATRIC	G52	Disorders of other cranial nerves		
NEUROPSYCHIATRIC	G53	Cranial nerve disorders in diseases classified elsewhere	352.9	Unspecified disorder of cranial nerves
NEUROPSYCHIATRIC	G70	Myasthenia gravis and other myoneural disorders		
NEUROPSYCHIATRIC	G71	Primary disorders of muscles		
NEUROPSYCHIATRIC	G723	Periodic paralysis	359.3	Periodic paralysis
NEUROPSYCHIATRIC	G724	Inflammatory myopathy, not elsewhere classified		
NEUROPSYCHIATRIC	G728	Other specified myopathies		
NEUROPSYCHIATRIC	G729	Myopathy, unspecified	359.9	Myopathy, unspecified
NEUROPSYCHIATRIC	G73	Disorders of myoneural junction and muscle in diseases classified elsewhere		
NEUROPSYCHIATRIC	G80	Cerebral palsy		
NEUROPSYCHIATRIC	G81	Hemiplegia		
NEUROPSYCHIATRIC	G82	Paraplegia and tetraplegia		
NEUROPSYCHIATRIC	G83	Other paralytic syndromes		
NEUROPSYCHIATRIC	G90	Disorders of autonomic nervous system		
NEUROPSYCHIATRIC	G91	Hydrocephalus		
NEUROPSYCHIATRIC	G938	Other specified disorders of brain		
NEUROPSYCHIATRIC	G939	Disorder of brain, unspecified	348.9	Unspecified condition of brain
NEUROPSYCHIATRIC	G95	Other diseases of spinal cord		
NEUROPSYCHIATRIC	G99	Other disorders of nervous system in diseases classified elsewhere		
NEUROPSYCHIATRIC	M471	Other spondylosis with myelopathy		
NEUROPSYCHIATRIC	Q00	Anencephaly and similar malformations		
NEUROPSYCHIATRIC	Q01	Encephalocele		
NEUROPSYCHIATRIC	Q02	Microcephaly	742.1	Microcephalus
NEUROPSYCHIATRIC	Q03	Congenital hydrocephalus		
NEUROPSYCHIATRIC	Q04	Other congenital malformations of brain		
NEUROPSYCHIATRIC	Q05	Spina bifida		
NEUROPSYCHIATRIC	Q06	Other congenital malformations of spinal cord		
NEUROPSYCHIATRIC	Q07	Other congenital malformations of nervous system		
NEUROPSYCHIATRIC	Q760	Spina bifida occulta	756.17	Spina bifida occulta
NEUROPSYCHIATRIC	F04	Organic amnesic syndrome, not induced by alcohol and other psychoactive substances	294.0	Amnestic disorder in conditions classified elsewhere
NEUROPSYCHIATRIC	F06	Other mental disorders due to brain damage and dysfunction and to physical disease		
NEUROPSYCHIATRIC	F07	Personality and behavioural disorders due to brain disease, damage and dysfunction		
NEUROPSYCHIATRIC	F09	Unspecified organic or symptomatic mental disorder	310.9	Unspecified nonpsychotic mental disorder following organic brain damage
NEUROPSYCHIATRIC	F102	Mental and behavioural disorders due to use of alcohol: Dependence syndrome		
NEUROPSYCHIATRIC	F106	Mental and behavioural disorders due to use of alcohol: Amnesic syndrome		
NEUROPSYCHIATRIC	F107	Mental and behavioural disorders due to use of alcohol: Residual and late-onset psychotic disorder		
NEUROPSYCHIATRIC	F112	Mental and behavioural disorders due to use of opioids: Dependence syndrome		
NEUROPSYCHIATRIC	F116	Mental and behavioural disorders due to use of opioids: Amnesic syndrome		
NEUROPSYCHIATRIC	F117	Mental and behavioural disorders due to use of opioids: Residual and late-onset psychotic disorder		
NEUROPSYCHIATRIC	F122	Mental and behavioural disorders due to use of cannabinoids: Dependence syndrome		
NEUROPSYCHIATRIC	F126	Mental and behavioural disorders due to use of cannabinoids: Amnesic syndrome		
NEUROPSYCHIATRIC	F127	Mental and behavioural disorders due to use of cannabinoids: Residual and late-onset psychotic disorder		
NEUROPSYCHIATRIC	F132	Mental and behavioural disorders due to use of sedatives or hypnotics: Dependence syndrome		
NEUROPSYCHIATRIC	F136	Mental and behavioural disorders due to use of sedatives or hypnotics: Amnesic syndrome		
NEUROPSYCHIATRIC	F137	Mental and behavioural disorders due to use of sedatives or hypnotics: Residual and late-onset psychotic disorder		
NEUROPSYCHIATRIC	F142	Mental and behavioural disorders due to use of cocaine: Dependence syndrome		
NEUROPSYCHIATRIC	F146	Mental and behavioural disorders due to use of cocaine: Amnesic syndrome		
NEUROPSYCHIATRIC	F147	Mental and behavioural disorders due to use of cocaine: Residual and late-onset psychotic disorder		
NEUROPSYCHIATRIC	F152	Mental and behavioural disorders due to use of other stimulants, including caffeine: Dependence syndrome		
NEUROPSYCHIATRIC	F156	Mental and behavioural disorders due to use of other stimulants, including caffeine: Amnesic syndrome		
NEUROPSYCHIATRIC	F157	Mental and behavioural disorders due to use of other stimulants, including caffeine: Residual and late-onset psychotic disorder		
NEUROPSYCHIATRIC	F162	Mental and behavioural disorders due to use of hallucinogens: Dependence syndrome		
NEUROPSYCHIATRIC	F166	Mental and behavioural disorders due to use of hallucinogens: Amnesic syndrome		
NEUROPSYCHIATRIC	F167	Mental and behavioural disorders due to use of hallucinogens: Residual and late-onset psychotic disorder		
NEUROPSYCHIATRIC	F172	Mental and behavioural disorders due to use of tobacco: Dependence syndrome		
NEUROPSYCHIATRIC	F176	Mental and behavioural disorders due to use of tobacco: Amnesic syndrome		
NEUROPSYCHIATRIC	F177	Mental and behavioural disorders due to use of tobacco: Residual and late-onset psychotic disorder		
NEUROPSYCHIATRIC	F182	Mental and behavioural disorders due to use of volatile solvents: Dependence syndrome		
NEUROPSYCHIATRIC	F186	Mental and behavioural disorders due to use of volatile solvents: Amnesic syndrome		
NEUROPSYCHIATRIC	F187	Mental and behavioural disorders due to use of volatile solvents: Residual and late-onset psychotic disorder		
NEUROPSYCHIATRIC	F192	Mental and behavioural disorders due to multiple drug use and use of other psychoactive substances: Dependence syndrome		
NEUROPSYCHIATRIC	F196	Mental and behavioural disorders due to multiple drug use and use of other psychoactive substances: Amnesic syndrome		
NEUROPSYCHIATRIC	F197	Mental and behavioural disorders due to multiple drug use and use of other psychoactive substances: Residual and late-onset psychotic disorder		
NEUROPSYCHIATRIC	F50	Eating disorders		
NEUROPSYCHIATRIC	F52	Sexual dysfunction, not caused by organic disorder or disease		
NEUROPSYCHIATRIC	F60	Specific personality disorders		
NEUROPSYCHIATRIC	F61	Mixed and other personality disorders		
NEUROPSYCHIATRIC	F62	Enduring personality changes, not attributable to brain damage and disease		
NEUROPSYCHIATRIC	F63	Habit and impulse disorders		
NEUROPSYCHIATRIC	F68	Other disorders of adult personality and behaviour		
NEUROPSYCHIATRIC	F70	Mild mental retardation	317	Mild intellectual disabilities
NEUROPSYCHIATRIC	F71	Moderate mental retardation	318.0	Moderate intellectual disabilities
NEUROPSYCHIATRIC	F72	Severe mental retardation	318.1	Severe intellectual disabilities
NEUROPSYCHIATRIC	F73	Profound mental retardation	318.2	Profound intellectual disabilities
NEUROPSYCHIATRIC	F78	Other mental retardation	319	Unspecified intellectual disabilities
NEUROPSYCHIATRIC	F79	Unspecified mental retardation	319	Unspecified intellectual disabilities
NEUROPSYCHIATRIC	F80	Specific developmental disorders of speech and language		
NEUROPSYCHIATRIC	F81	Specific developmental disorders of scholastic skills		
NEUROPSYCHIATRIC	F82	Specific developmental disorder of motor function	315.4	Developmental coordination disorder
NEUROPSYCHIATRIC	F83	Mixed specific developmental disorders		
NEUROPSYCHIATRIC	F84	Pervasive developmental disorders		
NEUROPSYCHIATRIC	F88	Other disorders of psychological development	315.8	Other specified delays in development
NEUROPSYCHIATRIC	F89	Unspecified disorder of psychological development	315.9	Unspecified delay in development
NEUROPSYCHIATRIC	F95	Tic disorders		
NEUROPSYCHIATRIC	F99	Mental disorder, not otherwise specified	300.9	Unspecified nonpsychotic mental disorder
NEUROPSYCHIATRIC	G20	Parkinson disease	332.0	Paralysis agitans
NEUROPSYCHIATRIC	G21	Secondary parkinsonism		
NEUROPSYCHIATRIC	G22	Parkinsonism in diseases classified elsewhere		
NEUROPSYCHIATRIC	G23	Other degenerative diseases of basal ganglia		
NEUROPSYCHIATRIC	F20	Schizophrenia		
NEUROPSYCHIATRIC	F22	Persistent delusional disorders	297.0	Paranoid state, simple
NEUROPSYCHIATRIC	F22	Persistent delusional disorders	297.1	Delusional disorder
NEUROPSYCHIATRIC	F22	Persistent delusional disorders	297.2	Paraphrenia
NEUROPSYCHIATRIC	F24	Induced delusional disorder	297.3	Shared psychotic disorder
NEUROPSYCHIATRIC	F25	Schizoaffective disorders		
NEUROPSYCHIATRIC	F28	Other nonorganic psychotic disorders	298.9	Unspecified psychosis
RESPIRATORY	J45	Asthma		
RESPIRATORY	J41	Simple and mucopurulent chronic bronchitis		
RESPIRATORY	J42	Unspecified chronic bronchitis	491.9	Unspecified chronic bronchitis
RESPIRATORY	J43	Emphysema		
RESPIRATORY	J44	Other chronic obstructive pulmonary disease		
RESPIRATORY	J47	Bronchiectasis		
RESPIRATORY	B909	Sequelae of respiratory and unspecified tuberculosis	137.0	Late effects of respiratory or unspecified tuberculosis
RESPIRATORY	E662	Extreme obesity with alveolar hypoventilation	278.03	Obesity hypoventilation syndrome
RESPIRATORY	J60	Coalworker pneumoconiosis	500	Coal workers’ pneumoconiosis
RESPIRATORY	J61	Pneumoconiosis due to asbestos and other mineral fibres	501	Asbestosis
RESPIRATORY	J62	Pneumoconiosis due to dust containing silica		
RESPIRATORY	J63	Pneumoconiosis due to other inorganic dusts		
RESPIRATORY	J64	Unspecified pneumoconiosis	505	Pneumoconiosis, unspecified
RESPIRATORY	J65	Pneumoconiosis associated with tuberculosis	505	Pneumoconiosis, unspecified
RESPIRATORY	J66	Airway disease due to specific organic dust		
RESPIRATORY	J67	Hypersensitivity pneumonitis due to organic dust		
RESPIRATORY	J684	Chronic respiratory conditions due to chemicals, gases, fumes and vapours	506.4	Chronic respiratory conditions due to fumes and vapors
RESPIRATORY	J701	Chronic and other pulmonary manifestations due to radiation	508.1	Chronic and other pulmonary manifestations due to radiation
RESPIRATORY	J703	Chronic drug-induced interstitial lung disorders	508.8	Respiratory conditions due to other specified external agents
RESPIRATORY	J704	Drug-induced interstitial lung disorders, unspecified	508.8	Respiratory conditions due to other specified external agents
RESPIRATORY	J84	Other interstitial pulmonary diseases		
RESPIRATORY	J92	Pleural plaque		
RESPIRATORY	J941	Fibrothorax	511.0	Pleurisy without mention of effusion or current tuberculosis
RESPIRATORY	J953	Chronic pulmonary insufficiency following surgery	518.52	Other pulmonary insufficiency, not elsewhere classified, following trauma and surgery
RESPIRATORY	J955	Postprocedural subglottic stenosis	997.39	Other respiratory complications
RESPIRATORY	J961	Chronic respiratory failure		
RESPIRATORY	J98	Other respiratory disorders		
RESPIRATORY	Q33	Congenital malformations of lung		
RESPIRATORY	Q34	Other congenital malformations of respiratory system		
RESPIRATORY	Z902	Acquired absence of lung [part of]	V45.76	Acquired absence of organ, lung
RESPIRATORY	Z942	Lung transplant status	V42.6	Postsurgical states following surgery of eye and adnexa
RESPIRATORY	Z943	Heart and lungs transplant status	V42.1	Postsurgical renal dialysis status
RESPIRATORY	Z943	Heart and lungs transplant status	V42.6	Postsurgical states following surgery of eye and adnexa
RESPIRATORY	Z963	Presence of artificial larynx	V43.81	Larynx replacement

## Appendix B

Collaborators of the PISCIS Cohort Study Group.
