# Supplementary material for: Comparative Analysis of Primary and Monovalent Booster SARS-CoV-2 Vaccination Coverage in Adults with and without HIV in Catalonia, Spain
Source: Vaccines (Basel). 2023 Dec 30;12(1):44. doi: 10.3390/vaccines12010044 (PMC10819920; doi:10.3390/vaccines12010044)
Supplement: Supplementary file 1 [file vaccines-12-00044-s001.zip › vaccines-2786783-Supplementary.pdf]

Supplementary Table S1: Factors associated with complete vaccine reception among HIV-negative participants in logistic regression analysis

Characteristic	Unvaccinated/ incomplete vaccination N = 33446	Complete vaccination N = 149854	OR (95% CI)	P value	aOR (95% CI)	P value
**Sex**[a]						
Male	28391 (84.9)	122229 (81.6)	1 (ref)		1 (ref)	
Female	5055 (15.1)	27625 (18.4)	1.27 (1.23,1.31)	<0.001	1.14 (1.1,1.19)	<0.001
**Age category, y**[b]						
16-30	5238 (15.7)	11112 (7.4)	1 (ref)		1 (ref)	
31-40	11294 (33.8)	32416 (21.6)	1.35 (1.3,1.41)	<0.001	1.32 (1.26,1.37)	<0.001
41-50	9560 (28.6)	47630 (31.8)	2.35 (2.26,2.44)	<0.001	1.84 (1.76,1.92)	<0.001
51-60	5651 (16.9)	42169 (28.1)	3.52 (3.37,3.67)	<0.001	2.27 (2.16,2.38)	<0.001
61-70	1389 (4.2)	12251 (8.2)	4.16 (3.9,4.43)	<0.001	2.12 (1.98,2.28)	<0.001
>70	314 (0.9)	4276 (2.9)	6.42 (5.7,7.23)	<0.001	2.73 (2.4,3.1)	<0.001
**Country of origin**[c]						
Spain	17488 (52.3)	117516 (78.4)	1 (ref)		1 (ref)	
Outside Spain	15916 (47.6)	32263 (21.5)	0.3 (0.29,0.31)	<0.001	0.36 (0.35,0.37)	<0.001
Missing	42 (0.1)	75 (0.1)				
**Socioeconomic deprivation**						
Least deprived	16170 (48.3)	74590 (49.8)	1 (ref)		1 (ref)	
Mildly deprived	6315 (18.9)	28725 (19.2)	0.99 (0.95,1.02)	0.393	0.85 (0.82,0.88)	<0.001
Moderately/severely deprived	10207 (30.5)	43113 (28.8)	0.92 (0.89,0.94)	<0.001	0.86 (0.83,0.89)	<0.001
Missing	754 (2.3)	3426 (2.3)				
**Number of comorbidities**						
0	19309 (57.7)	62821 (41.9)	1 (ref)		1 (ref)	
1	7106 (21.2)	34853 (23.3)	1.51 (1.46,1.55)	<0.001	1.24 (1.2,1.29)	<0.001
2	3602 (10.8)	22201 (14.8)	1.89 (1.82,1.97)	<0.001	1.43 (1.37,1.5)	<0.001
3	1824 (5.5)	14137 (9.4)	2.38 (2.26,2.51)	<0.001	1.67 (1.58,1.77)	<0.001
≥4	1605 (4.8)	15842 (10.6)	3.03 (2.87,3.2)	<0.001	1.89 (1.78,2.01)	<0.001
**Previous SARS-CoV-2 diagnosis**						
No	23011 (68.8)	137650 (91.9)	1 (ref)		1 (ref)	
Yes	10435 (31.2)	12204 (8.1)	0.2 (0.19,0.2)	<0.001	0.18 (0.17,0.18)	<0.001

Abbreviations: OR, odds ratio; aOR, adjusted odds ratio; SARS-CoV-2, PLWH, people living with HIV; SARS-CoV-2, severe acute respiratory syndrome coronavirus 2; IQR, interquartile range; PWID, people who inject drugs; MSM, men who have sex with men; ART, antiretroviral therapy.

[a] Sex as assigned birth.

[b] Age for all patients was as at January 1, 2021.

[c] Country of origin was as indicated by the Public Data Analysis for Health Research and Innovation Program of Catalonia (PADRIS) recorded as Spanish or Non-Spanish.

Supplementary Table S2: Factors associated with booster vaccine reception among HIV-negative participants in logistic regression analysis

Characteristic	Unvaccinated/ incomplete vaccination N = 55345	Complete vaccination N = 94509	OR (95% CI)	P value	aOR (95% CI)	P value
**Sex[a]**						
Male	46025 (83.2)	76204 (80.6)	1 (ref)		1 (ref)	
Female	9320 (16.8)	18305 (19.4)	1.19 (1.15,1.22)	<0.001	1.07 (1.04,1.11)	<0.001
**Age category, y[b]**						
16-30	7040 (12.7)	4072 (4.3)	1 (ref)		1 (ref)	
31-40	16903 (30.5)	15513 (16.4)	1.59 (1.52,1.66)	<0.001	1.61 (1.54,1.68)	<0.001
41-50	18292 (33.1)	29338 (31)	2.77 (2.66,2.89)	<0.001	2.76 (2.64,2.88)	<0.001
51-60	11103 (20.1)	31066 (32.9)	4.84 (4.63,5.06)	<0.001	4.71 (4.5,4.94)	<0.001
61-70	1678 (3)	10573 (11.2)	10.89 (10.21,11.62)	<0.001	10.15 (9.47,10.87)	<0.001
>70	329 (0.6)	3947 (4.2)	20.74 (18.42,23.36)	<0.001	18.23 (16.08,20.66)	<0.001
**Country of origin[c]**						
Spain	39354 (71.1)	78162 (82.7)	1 (ref)		1 (ref)	
Outside Spain	15959 (28.8)	16304 (17.3)	0.51 (0.5,0.53)	<0.001	0.73 (0.71,0.75)	<0.001
Missing	32 (0.1)	43 (0)				
**Socioeconomic deprivation**						
Least deprived	26673 (48.2)	47917 (50.7)	1 (ref)		1 (ref)	
Mildly deprived	10577 (19.1)	18148 (19.2)	0.96 (0.93,0.98)	0.001	0.8 (0.77,0.82)	<0.001
Moderately/severely deprived	16843 (30.4)	26270 (27.8)	0.87 (0.85,0.89)	<0.001	0.77 (0.75,0.79)	<0.001
Missing	1252 (2.3)	2174 (2.3)				
**Number of comorbidities**						
0	26848 (48.5)	35973 (38.1)	1 (ref)		1 (ref)	
1	13431 (24.3)	21422 (22.7)	1.19 (1.16,1.22)	<0.001	1 (0.97,1.03)	0.926
2	7466 (13.5)	14735 (15.6)	1.47 (1.43,1.52)	<0.001	1.08 (1.04,1.11)	<0.001
3	4143 (7.5)	9994 (10.6)	1.8 (1.73,1.87)	<0.001	1.15 (1.1,1.2)	<0.001
≥4	3457 (6.2)	12385 (13.1)	2.67 (2.57,2.79)	<0.001	1.31 (1.25,1.37)	<0.001
**Previous SARS-CoV-2 diagnosis**						
No	47247 (85.4)	90403 (95.7)	1 (ref)		1 (ref)	
Yes	8098 (14.6)	4106 (4.3)	0.26 (0.25,0.28)	<0.001	0.23 (0.22,0.24)	<0.001

Abbreviations: OR, odds ratio; aOR, adjusted odds ratio; SARS-CoV-2, PLWH, people living with HIV; SARS-CoV-2, severe acute respiratory syndrome coronavirus 2; IQR, interquartile range; PWID, people who inject drugs; MSM, men who have sex with men; ART, antiretroviral therapy.

[a] Sex as assigned birth.

[b] Age for all patients was as at January 1, 2021.

[c] Country of origin was as indicated by the Public Data Analysis for Health Research and Innovation Program of Catalonia (PADRIS) recorded as Spanish or Non-Spanish.

Supplementary Table S3: Factors associated with complete vaccine reception among PLWH in logistic regression analysis

**Characteristic**	**Unvaccinated/ incomplete vaccination N = 3995**	**Complete vaccination N = 14335**	**OR (95% CI)**	**P value**	**aOR (95% CI)**	**P value**
**Sex[a]**						
Male	3262 (81.7)	11800 (82.3)	1 (ref)		1 (ref)	
Female	733 (18.3)	2535 (17.7)	0.96 (0.87,1.05)	0.332	0.82 (0.75,0.9)	<0.001
**Age category, y[b]**						
16-30	582 (14.6)	1053 (7.3)	1 (ref)		1 (ref)	
31-40	1213 (30.4)	3158 (22)	1.44 (1.28,1.62)	<0.001	1.25 (1.11,1.42)	<0.001
41-50	1197 (30)	4522 (31.5)	2.09 (1.85,2.35)	<0.001	1.54 (1.36,1.75)	<0.001
51-60	760 (19)	4022 (28.1)	2.92 (2.57,3.32)	<0.001	1.89 (1.64,2.18)	<0.001
61-70	191 (4.8)	1173 (8.2)	3.39 (2.83,4.08)	<0.001	2.05 (1.68,2.5)	<0.001
>70	52 (1.3)	407 (2.8)	4.33 (3.19,5.87)	<0.001	2.52 (1.81,3.49)	<0.001
**Country of origin[c]**						
Spain	1833 (45.9)	8833 (61.6)	1 (ref)		1 (ref)	
Outside Spain	2160 (54.1)	5502 (38.4)	0.53 (0.49,0.57)	<0.001	0.7 (0.64,0.75)	<0.001
Missing	2 (0.1)	0 (0)				
**Socioeconomic deprivation**						
Least deprived	2161 (54.1)	6915 (48.2)	1 (ref)		1 (ref)	
Mildly deprived	642 (16.1)	2862 (20)	1.39 (1.26,1.54)	<0.001	1.21 (1.09,1.34)	<0.001
Moderately/severely deprived	1107 (27.7)	4225 (29.5)	1.19 (1.1,1.29)	<0.001	1.06 (0.97,1.16)	0.173
Missing	85 (2.1)	333 (2.3)				
**Number of comorbidities**						
0	1545 (38.7)	3479 (24.3)	1 (ref)		1 (ref)	
1	914 (22.9)	3169 (22.1)	1.54 (1.4,1.69)	<0.001	1.34 (1.21,1.48)	<0.001
2	606 (15.2)	2693 (18.8)	1.97 (1.77,2.2)	<0.001	1.56 (1.39,1.75)	<0.001
3	395 (9.9)	1992 (13.9)	2.24 (1.98,2.53)	<0.001	1.63 (1.43,1.86)	<0.001
≥4	535 (13.4)	3002 (20.9)	2.49 (2.23,2.78)	<0.001	1.64 (1.45,1.87)	<0.001
**Previous SARS-CoV-2 diagnosis**						
No	3070 (76.8)	12806 (89.3)	1 (ref)		1 (ref)	
Yes	925 (23.2)	1529 (10.7)	0.4 (0.36,0.43)	<0.001	0.42 (0.38,0.46)	<0.001

Abbreviations: OR, odds ratio; aOR, adjusted odds ratio; SARS-CoV-2, PLWH, people living with HIV; SARS-CoV-2, severe acute respiratory syndrome coronavirus 2; IQR, interquartile range; PWID, people who inject drugs; MSM, men who have sex with men; ART, antiretroviral therapy.

[a] Sex as assigned birth.

[b] Age for all patients was as at January 1, 2021.

[c] Country of origin was as indicated by the Public Data Analysis for Health Research and Innovation Program of Catalonia (PADRIS) recorded as Spanish or Non-Spanish.

Supplementary table S4: Factors associated with booster vaccine reception among PLWH in logistic regression analysis

	**Unvaccinated/ incomplete vaccination**	**Complete vaccination**	**OR (95% CI)**	**P value**	**aOR (95% CI)**	**P value**
**Characteristic**	**N = 4512**	**N = 9823**				
**Sex[a]**						
Male	3572 (79.2)	8228 (83.8)	1 (ref)		1 (ref)	
Female	940 (20.8)	1595 (16.2)	0.74 (0.67,0.81)	<0.001	0.62 (0.56,0.68)	<0.001
**Age category, y[b]**						
16-30	596 (13.2)	457 (4.7)	1 (ref)		1 (ref)	
31-40	1371 (30.4)	1787 (18.2)	1.7 (1.48,1.96)	<0.001	1.74 (1.51,2.02)	<0.001
41-50	1405 (31.1)	3117 (31.7)	2.89 (2.52,3.32)	<0.001	2.96 (2.56,3.43)	<0.001
51-60	925 (20.5)	3097 (31.5)	4.37 (3.79,5.03)	<0.001	4.45 (3.8,5.2)	<0.001
61-70	174 (3.9)	999 (10.2)	7.49 (6.12,9.16)	<0.001	7.62 (6.12,9.49)	<0.001
>70	41 (0.9)	366 (3.7)	11.64 (8.25,16.44)	<0.001	11.24 (7.82,16.16)	<0.001
**Country of origin[c]**						
Spain	2462 (54.6)	6371 (64.9)	1 (ref)		1 (ref)	
Outside Spain	2050 (45.4)	3452 (35.1)	0.65 (0.61,0.7)	<0.001	0.91 (0.84,0.99)	0.021
Missing	0 (0)	0 (0)				
**Socioeconomic deprivation**						
Least deprived	2104 (46.6)	4811 (49)	1 (ref)		1 (ref)	
Mildly deprived	877 (19.4)	1985 (20.2)	0.99 (0.9,1.09)	0.833	0.87 (0.79,0.96)	0.006
Moderately/severely deprived	1426 (31.6)	2799 (28.5)	0.86 (0.79,0.93)	<0.001	0.79 (0.72,0.86)	<0.001
Missing	105 (2.3)	228 (2.3)				
**Number of comorbidities**						
0	1320 (29.3)	2159 (22)	1 (ref)		1 (ref)	
1	1061 (23.5)	2108 (21.5)	1.21 (1.1,1.34)	<0.001	1.02 (0.91,1.13)	0.767
2	857 (19)	1836 (18.7)	1.31 (1.18,1.46)	<0.001	0.99 (0.88,1.11)	0.854
3	576 (12.8)	1416 (14.4)	1.5 (1.33,1.69)	<0.001	0.98 (0.86,1.12)	0.743
≥4	698 (15.5)	2304 (23.5)	2.02 (1.81,2.25)	<0.001	1.12 (0.98,1.27)	0.093
**Previous SARS-CoV-2 diagnosis**						
No	3655 (81)	9151 (93.2)	1 (ref)		1 (ref)	
Yes	857 (19)	672 (6.8)	0.31 (0.28,0.35)	<0.001	0.32 (0.28,0.36)	<0.001

Abbreviations: OR, odds ratio; aOR, adjusted odds ratio; SARS-CoV-2, PLWH, people living with HIV; SARS-CoV-2, severe acute respiratory syndrome coronavirus 2; IQR, interquartile range; PWID, people who inject drugs; MSM, men who have sex with men; ART, antiretroviral therapy.

[a] Sex as assigned birth.

[b] Age for all patients was as at January 1, 2021.

[c] Country of origin was as indicated by the Public Data Analysis for Health Research and Innovation Program of Catalonia (PADRIS) recorded as Spanish or Non-Spanish.
